# How might magnetic secular variation impact avian philopatry?

**DOI:** 10.1007/s00359-021-01533-y

**Published:** 2022-02-12

**Authors:** Joe Wynn, Oliver Padget, Joe Morford, Paris Jaggers, Katrina Davies, Emma Borsier, Tim Guilford

**Affiliations:** Department of Zoology, Oxford Navigation Group, 11a Mansfield Road, Oxford, OX1 3SZ Oxfordshire UK

**Keywords:** Magnetoreception, Navigation, Magnetic map, Secular variation, Bird migration

## Abstract

A tendency to return to the natal/breeding site, ‘philopatry’, is widespread amongst migratory birds. It has been suggested that a magnetic ‘map’ could underpin such movements, though it is unclear how a magnetic map might be impacted by gradual drift in the Earth’s magnetic field (‘secular variation’). Here, using the International Geomagnetic Reference Field, we quantified how secular variation translates to movement in the implied positions at which combinations of different magnetic cues (inclination, declination and intensity) intersect, noting that the magnitude of such movements is determined by the magnitude of the movements of each of the two isolines, and the angle between their movement vectors. We propose that magnetic parameters varying in a near-parallel arrangement are unlikely to be used as a bi-coordinate map during philopatry, but that birds could use near-orthogonal magnetic gradient cues as a bi-coordinate map if augmented with navigation using more local cues. We further suggest that uni-coordinate magnetic information could also provide a philopatry mechanism that is substantially less impacted by secular variation than a bi-coordinate ‘map’. We propose that between-year shifts in the position of magnetic coordinates might provide a priori predictions for changes in the breeding sites of migratory birds.

**Supplementary Information:**

The online version contains supplementary material available at 10.1007/s00359-021-01533-y.

## Introduction

Every year, many billions of migratory birds from across the avian phylogenetic tree return from their wintering sites, in doing so often pinpointing the location of their breeding site with remarkable accuracy (Newton and Brockie 2008). Such migrations regularly cover thousands of kilometres (e.g. Delmore et al. 2020) are often trans-equatorial (e.g. Guilford et al. 2009) and sometimes involve migrating from one polar latitude to the other (e.g. Egevang et al. 2010). Such journeys are typically thought to require both a compass to provide directional cues and a map to provide positional information (Kramer 1950), though the sensory basis of such long-distance map-based navigation is unclear, with very long-distance gradient cues suggested as a potential mechanism by which birds could precisely return to their natal or breeding sites (a process known as ‘philopatry’). Such cues could be learnt prior to departure and, subsequently, could be used to target the natal site over very long distances (Baker 1978; Lohmann et al. 2008). The main candidates for such global navigational gradients are thought to be olfactory (Padget et al. 2017; Pollonara et al. 2015; Gagliardo 2013; Gagliardo et al. 2013) or, alternatively, geomagnetic (Mouritsen 2018, 2003; Holland 2014).

Geomagnetic cues might, in principle, make excellent gradient cues for long-distance navigation owing to their generally ubiquitous availability (subject to magnetic anomalies and space weather; Dennis et al. 2007; Wiltschko et al. 2009; Granger et al. 2020) and the very long distances over which they vary (meaning that position relative to a gradient can be ascertained on a global scale). There have been five components of the Earth’s magnetic field that are considered to be navigationally useful in animals: inclination, the ‘dip angle’ between the Earth’s magnetic field and the Earth’s surface; declination, the angle between true and magnetic north; intensity, the overall strength of the Earth’s magnetic field vector and as the horizontal and vertical components of the magnetic field vector (for reviews, see Holland 2014; Mouritsen 2018). There exists evidence for the use of such cues in both avian and non-avian taxa, notably sea turtles (Lohmann et al. 2012; Lohmann and Lohmann 1994, 1996), fish (Naisbett-Jones et al. 2017; Putman et al. 2013, 2014) and songbirds (Passeriformes; Chernetsov et al. 2020; Chelonidae; Chernetsov et al. 2017; Kishkinev et al. 2021). In songbirds, inclination has been suggested to both act as a compass cue and provide positional information, with the mirror image pattern of inclination across the equator providing compass and position cues potentially robust to inversions of the Earth’s magnetic field (Wiltschko and Wiltschko 1972, 1992; Beason 1992; Fransson et al. 2001), whilst declination and/or intensity have been postulated to also provide position (Kishkinev et al. 2015, 2021; Chernetsov et al. 2017, 2020; Pakhomov et al. 2018).

However, a single magnetic gradient cue positions a bird with respect to only one spatial dimension. In some taxa, for example sea turtles or pelagic seabirds, topographic constraints (e.g. the edge of a land mass) may allow for the use of a single coordinate system with topography providing the second dimension required for bi-coordinate positioning (Wynn et al. 2020; Brothers and Lohmann 2018). Magnetic cues could, however, potentially provide both the longitude and latitude of a breeding site, with the intersection of two or more magnetic isolines denoting the two-dimensional location of the breeding site (Padget et al. 2019; Holland 2014; Lohmann et al. 2007). Such bi-coordinate magnetic ‘maps’ have been suggested to underly philopatry in certain avian taxa, specifically in songbirds (e.g. Pakhomov et al. 2018).

One of the primary limitations of magnetic cues regarding philopatry could be secular variation in magnetic cues (Putman and Lohmann 2008); year-on-year variation in the Earth’s magnetic field that causes the geographic position occupied by specific magnetic parameters to shift. The shifts in a given cue are very slight, typically a few kilometres per year (Putman and Lohmann 2008). However, if the geographic location of the natal site were to be represented using bi-coordinate magnetic information, it is unclear what the cumulative effects of secular variation in two cues would mean for movement in the supposed position of the natal/breeding site.

Here, we investigated how secular variation in magnetic cues translates into between-year variation in the geographic position occupied by specific cue values. We used the International Geomagnetic Reference Field 12 (IGRF 12; Thebault et al. 2015), a mathematical model of the Earth’s magnetic field over the last century, to quantify the distance between the site previously occupied by specific magnetic parameter values and the site subsequently occupied by the same values. Specifically, we looked at how the geographic points indicated by the intersect coordinates of inclination/intensity, inclination/declination and intensity/declination isolines varied between years in three parts of the globe suggested to be well-suited to magnetic navigation: central North America, Europe and Central Asia (Bostrom et al. 2012).

## Methods

All statistics were calculated in R (R Team 2017).

### International Geomagnetic Reference Field (IGRF) modelling

**Fig. 1:**
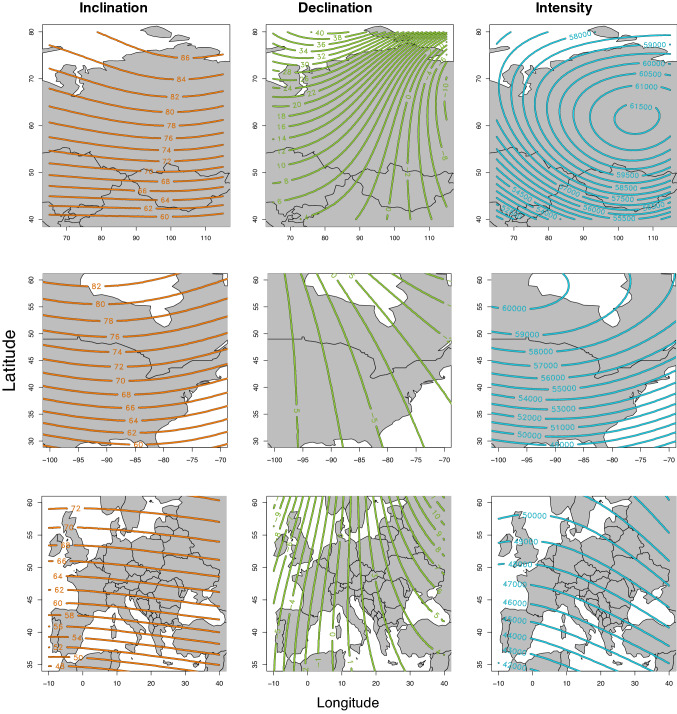
Magnetic isoline positions in the year 2000. For each region of the Earth examined, Asia (top), North America (centre) and Europe (bottom), isolines for the year 2000 are plotted for (from left) inclination, declination and intensity

Yearly magnetic values, averaged from 12 dates between May and August, the timeframe over which Northern Hemisphere migratory birds are likely to reside at the natal/breeding site, were derived from the International Geomagnetic Reference Field 12 (‘IGRF’) for each of inclination, intensity and declination for sites across North America, Europe and Asia at a spatial resolution of 0.05° × 0.05° (see Fig. 1 for isoline arrangements in a given year). For North America, values were extracted from the IGRF across a latitudinal range of 25°–65° and a longitudinal range of − 10° to 40°; for Europe, values were extracted for a latitudinal range of 25°–65° and a longitudinal range of − 115° to − 65°; and for Asia values were extracted for a latitudinal range of 40°–80° and a longitudinal range of 65°–115°.

**Fig. 2:**
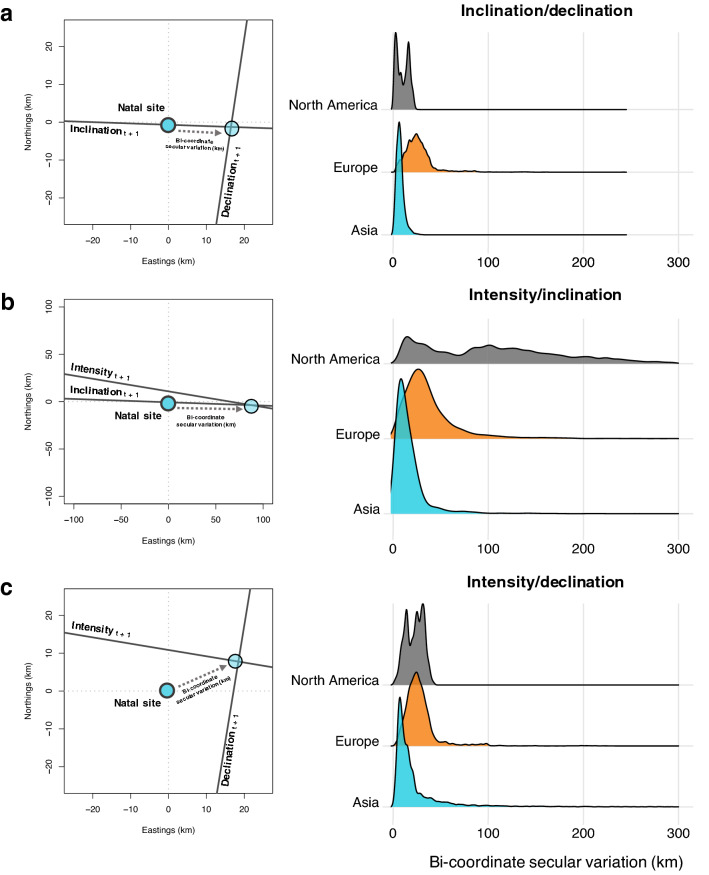
The effect of secular variation on the position of different isoline combinations. Empirical examples of how the intersect of each pair of isolines can be located in successive years (left) and the distance moved by the intersect of two isolines (right) (‘Bi-coordinate secular variation’) in successive years for each of **a** inclination/declination, **b** intensity/inclination and **c** intensity/declination. For each set of isolines, the distance moved is displayed as a density curve (bandwidth = 5 km), with the height of a given curve representing the probability of a bird moving that distance. The area under each curve is, therefore, constant, which allows for between-curve comparisons, and larger peaks in the curve denote greater frequency amongst the observed data

For each of North America, Europe and Asia we selected 100 geographic positions at random where, over the years 1900–2000, yearly values for inclination, declination and intensity were calculated using the IGRF. Based on these values, between-year variation in the positions occupied by specific magnetic isolines could be calculated. To do this the magnetic parameter values representing a given site were calculated for a given year, and the isoline representing the same value was isolated in the next year. This was repeated for each of inclination, intensity and declination. Because the focal value from the previous year is not necessarily present in the subsequent year, we defined the subsequent-year isoline for inclination and declination as points within 0.01° of the focal value and for intensity we defined the isoline as points within 10nT of the focal value. For example, if a site had an inclination value of 65°, a declination value of 5° and an intensity value of 49,500nT, the geographic positions of the 65° (± 0.01°) inclination isoline, the 5° declination isoline (± 0.01°) and the 49,500nT (± 10nT) intensity isoline would be isolated in the following year. Once the location of the isolines in the subsequent year had been established, the location of the intersects of these isolines in the next year were then calculated. Finally, the distance between the site previously occupied by the intersect of specific parameter values and the site subsequently occupied by the same values were calculated. This is summarised in Fig. 2.

### Modelling the factors that predict changes in magnetic bi-coordinate position

We derived an equation to predict the geographic distance moved by specific magnetic coordinates (i.e. the intersection between the isolines in two magnetic parameters sensed/learnt at that geographic site the year previously) between years. This uses the vector moved by each of two isolines between years, and the angle between these vectors. Vectors of isoline movement for a given magnetic parameter can be approximated at a focal site as the vector between the focal site and the closest site with same magnetic parameter value in the following year. The position of the intersect (and hence, the distance moved) between these two isolines can then be determined from the vectors by making the following three assumptions: (1) that the curvature of Earth is of negligible importance, (2) that the angle between two isolines does not change substantially between years and (3) that, over short distances, a given magnetic isoline could be approximated as a straight line, perpendicular to its vector of movement between years. We considered these assumptions valid since the distance over which magnetic isolines moved was typically < 500 km, a scale over which the curvature of earth and the non-linear nature magnetic isolines appeared negligible (see Fig. 1), and the angle between two isolines was observed to change negligibly between years (see Fig. 1).

The equation giving the approximate geographic distance moved by an intersection of two isolines between years is given below. ${d}_{x}$ and ${d}_{y}$ denote the vector magnitudes of the isoline movements of the two magnetic parameters in question, and *θ* denotes the angle between these vectors. The equation was derived through modelling each isoline with a linear equation, and solving for their intersection.

1 $${d}_{\mathrm{overall}}= \sqrt{{{d}_{x}}^{2}+{\left(\frac{{{d}_{y}- d}_{x}\mathrm{cos}\theta }{\mathrm{sin}\theta }\right)}^{2}}$$

Using this equation we can, therefore, vary the angle between pairs of isolines in our model (*θ*) to investigate how this impacts the geographic position that would be arrived at if navigating to those magnetic coordinates (i.e. that intersection). Further, by simultaneously varying the angle between isolines and the magnitude of each isoline’s movement, we can manipulate isoline vectors so as to vary the direction of an isolines’ vector movement relative to another isoline. This allows us to investigate the extent to which this distance moved by isoline intersect is affected by both the angle at which the isolines intersected, and the distance and direction moved by each isoline.

## Results

### Between-year variation in bi-coordinate magnetic position

Using the IGRF, we quantified the extent to which the intersect of specific magnetic isolines moved between years. We found that the median geographic distance moved by the magnetic coordinate formed by inclination and declination between years was 11.0 km (± 0.204 km [bootstrapped 95% CI]). For a coordinate comprising declination and intensity measurements, the median geographic distance moved was 21.4 km (± 0.211 km) between years, and, finally, for a coordinate comprising inclination and intensity, a median of 28.4 km (± 0.211 km; see Fig. 2; see supplementary material for more information).

### Modelling the factors that predict changes in bi-coordinate magnetic position

**Fig. 3:**
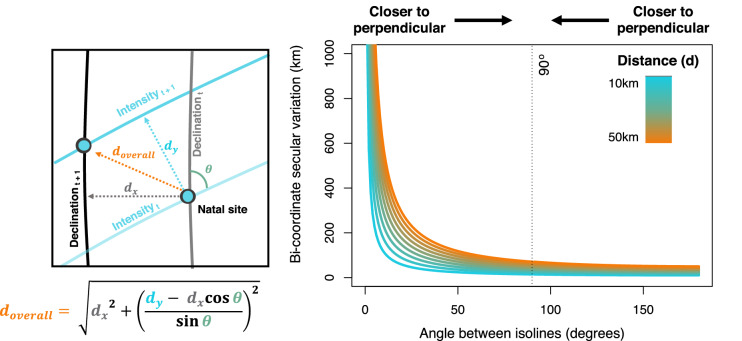
Modelling the effects on bi-coordinate secular variation of the distance moved by isolines and the angular difference between isolines. Schematic showing how the distance moved by two isolines (left) (in this example intensity and declination) and the angle between the same isolines are calculated, with the equation used to discern bi-coordinate movement in position based on this information shown below. Colours are consistent between the diagram and equation, with the angle theta denoting the angle between isoline vector movements. The effect of the angle between two isolines on bi-coordinate secular variation (right). In these simulations, isolines are set to move the same distance, with distances between 10 and 50 km included and colour-coded with blue lines representing shorter distances and orange lines representing longer distances. Here, the angle between isolines is shown on a 0°–180° scale, with 90° representing perpendicular isoline vector movements, 180° representing parallel and aligned isoline vector movements and 0° representing parallel and opposing isoline vector movements

Using Eq. (1), we investigated how variation in the position denoted by the intersect of two isolines varies with (a) the distance moved by the isolines in question (${d}_{x}$ and ${d}_{y}$) and (b) the acute angle between these isolines (θ). We found that the effect of the angle between isolines was contingent on whether isoline vector movements were aligned or opposed. When isolines movement vectors were aligned we found that smaller angles (i.e. near-parallel angles) reduced the effect of secular variation on the distance moved by the isoline intersect, with 10 km of movement per isoline translating to 10 km bi-coordinate movement when isolines were parallel and 14.4 km bi-coordinate movement when isolines were perpendicular (see Fig. 3). Conversely, we found that if the vectors of isoline movement were opposed then larger angles (i.e. near-perpendicular angles) reduced the effect of secular variation, with 10 km of isoline movement causing 1146 km of bi-coordinate movement when isolines were almost-parallel and 14.4 km of bi-coordinate movement when isolines were perpendicular (see Fig. 3). In all instances, we found that the greater the movement of isolines, the greater the movement of their intersect position, though the isoline intersect’s movement was greatest in instances where near-parallel isolines were moving in opposite directions (see Fig. 3).

### The effect of the acute angle between isolines on the magnitude and variance of bi-coordinate secular variation

**Fig. 4:**
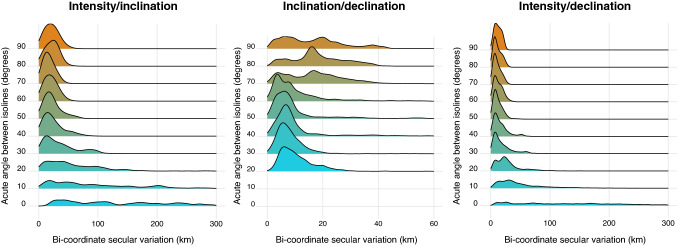
The observed effect of the angle between isolines on bi-coordinate secular variation in different coordinate systems. The effect of the angle between isolines on bi-coordinate secular variation (from left) intensity/inclination, inclination/declination and intensity/declination. For each set of isolines the distance moved is displayed as a density curve (bandwidth = 5 km), with the height of a given curve at a given point representing the probability of a bird moving that distance. The area under each curve is, therefore, constant, which allows for between-curve comparisons, and larger peaks in the curve denote greater frequency amongst the observed data

**Fig. 5:**
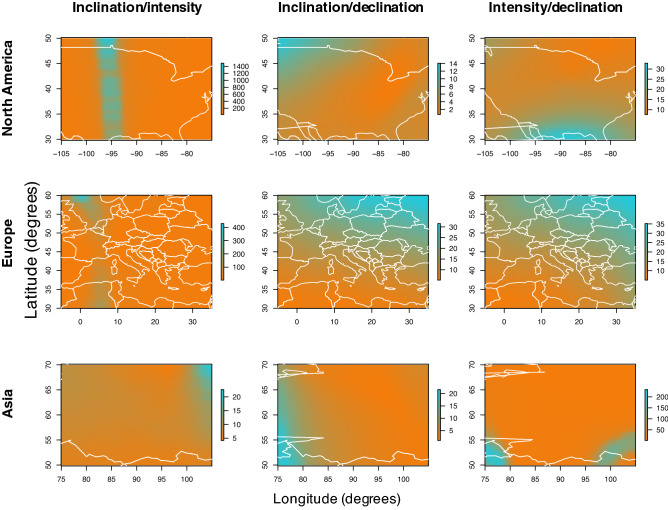
Secular variation in bi-coordinate position between 1900 and 1901. As modelled with the IGRF, the estimated change in the bi-coordinate intersect position (see Fig. 2) represented by isolines of (left) inclination/declination, (centre) inclination/intensity and (right) intensity/declination for North America (top), Europe (centre) and Asia (bottom). All values are given in kilometres, and the scale of each plot is independent owing the several orders of magnitude difference between the highest and lowest values

Based on the predictions of our model, we used the IGRF to investigate whether the angle between isolines explained variance in the distance moved by intersect positions between years. We found that the angle between two isolines was a significant predictor of the movement of the position denoted by the intersect of isolines for all pairs of cues investigated (inclination/intensity; LM, *F* = 1324, *p* < 0.00001; inclination/declination; LM, *F* = 671, *p* < 0.00001; intensity/declination; *F* = 1640, LM, *p* < 0.00001). For both inclination/intensity (gradient = − 3.95 ± 0.22) and declination/intensity (gradient = − 1.4 ± 0.068), we found that isolines became closer to parallel (i.e. the angle between isolines decreased) the movement of the isoline intersect was greater. In contrast, we found that movement in the site denoted by the intersect of inclination/declination isolines reduced slightly as isolines were closer to parallel (gradient = 0.09 ± 0.0071; see Fig. 4). We also found that differences in the angle between isolines could cause very large differences in the movements of isoline-intersect positions even within relatively small areas. For example, within Europe the positions denoted by inclination/intensity isolines intersects moved a median of 95 km in the UK but an average of 28 km in Poland, meaning that in principle even within the breeding range of a single species (e.g. the Eurasian reed warbler; *Acrocephalus scirpaceus*) the effect of secular variation might vary greatly (see Fig. 5).

In addition to finding that the angle between isolines affected the distance between the geographic positions denoted by the shifting isoline intersect in consecutive years, we also found that as the angle between isolines reduced, the variance in the effect of secular variation increased. We found that, when the acute angle between intensity/inclination isolines was < 20°, the standard deviation in the distance moved by the intersection was 613 km whilst, when the acute angle between two isolines was > 70°, the standard deviation in the distance moved was 5.96 km (see Fig. 4). We found that this was also the case when considering both intensity/declination (where a standard deviation of 606 km was observed when the angle between isolines was < 20° and a standard deviation of 11.5 km was observed when the angle was > 70°) and, to a lesser extent, inclination/declination (where a standard deviation of 17.2 km was observed when the angle between isolines was < 20° and a standard deviation of 9.06 km was observed when the angle was > 70°; see Fig. 4). This implied that as the angle between isolines decreased, not only did the isoline intersect movement distance increase but, additionally, became more variable.

## Discussion

Using the IGRF we found measurable between-year movements in position as denoted using the intersect of isolines of inclination/declination, intensity/declination and inclination/intensity across all areas sampled. We found that the extent to which magnetic coordinates moved over geographic space was dependent on the angle between isolines, the extent to which isolines moved and whether isolines were moving in the same or different directions. We further found that variance in the extent to which isoline intersects moved also changed with the angle between isolines. Below, we outline why near-parallel isolines have seemingly limited utility to achieve philopatry, and propose mechanisms by which migratory animals might counter the effects of secular variation. We further suggest that the empirical comparison of distances moved between years by migratory birds to those predicted under a model of magnetic bi-coordinate navigation may be informative when investigating which sensory cues underpin philopatry and, more specifically, how these cues combine to give an indication of position in two dimensions (Putman et al. 2013; Brothers and Lohmann 2015; Wynn et al. 2020).

Near-parallel gradient cues have been suggested to be of limited navigational use to animals for a variety of reasons, most notably owing to the necessarily high resolution with which cues would have to be discerned to be useful (Bostrom et al. 2012; Akesson and Alerstam 1998). Less accurate sensors would necessarily reduce certainty in any positional estimates, and hence the area within which the target could be found increases. Additionally, it has been questioned whether near-parallel gradient cues could be used by animals during straight-line orientation if animals were constrained to cognitive processing that interpreted cues as if they were perpendicular (Benhamou 2003). However, even if an animal were able to detect magnetic cues with perfect accuracy, and process them correctly, it would seem that secular variation makes near-parallel magnetic gradients extremely difficult to use for precise natal homing. This is owing not only to large between-year movement in the position denoted by the intercept but also owing to the large year-on-year variance in the distance moved by isoline intercept positions. This variance, caused by the differing effects of the angle between isolines when isolines move movement vectors are aligned or opposed, makes field movement erratic and seemingly difficult to account for. This would be further exacerbated by inaccuracies in any putative magnetoreceptor, which in turn would increase uncertainty in the position of the target. We suggest, therefore, that it is unlikely that birds could rely on pairs of magnetic cues that vary through space along near-parallel gradients when re-locating a natal/breeding site.

Isolines of inclination/intensity and declination/intensity run largely non-orthogonally across our sample areas and we might, therefore, predict that they are of limited use during philopatry. However, we found that, in most parts of the globe, inclination and declination formed a more perpendicular grid. Further, movement in the inclination/declination-denoted position reduced as isolines became closer to parallel, hence even as isolines became closer to parallel the effect of secular variation was limited (see Fig. 4). Of the candidate bi-coordinate navigation hypotheses inclination/declination has, therefore, perhaps the greatest overall utility when indicating a geographic position. Given that this is the case, further experimentation regarding the use of geomagnetic declination as a spatial cue seems essential.

However, we found that even when considering near-perpendicular cues there are movements in the position denoted by specific isoline intersects, likely sufficient to require augmentation from other navigational mechanisms, in the position of specific magnetic coordinates owing to secular variation. Such movements, whilst an order of magnitude smaller than the movement of near-parallel cues, could nonetheless impact philopatry amongst birds. For example, random movements of the geographic location of an isoline intersect of a magnitude between 0 and 20 km could mean that the intersect value could occur anywhere within a 20 km radius (an area of 1256 km^2^). It would seem, therefore, unlikely that magnetic parameters alone are sufficient to perform faithful philopatry. It has been suggested that return migration might comprise several fairly distinct ‘phases’, with long-distance navigation underpinned by spatial gradient cues and locale specific landmark cues thought to underpin precise local-scale homing (for a review see Mouritsen 2018). Birds could, therefore, counter the effects of secular variation by having a sufficiently large familiar area. Indeed there is some evidence that prior to first migration young songbirds make night-time forays away from their natal site (e.g. Mukhin et al. 2005; Baker 1978), and such trips could be used to parameterise an appropriately large familiar area map.

Additionally, or alternatively, birds could use magnetic cues to determine position in only one dimension, relying on other cues to give the second. This would, necessarily, limit the multiplicative effects of secular variation on multiple cues. One mechanism by which birds could limit their exposure to secular variation would be to, as is seemingly the case in sea turtles, use topographic barriers (alongside uni-coordinate magnetic information) to position themselves with regards to both longitude and latitude (Putman and Lohmann 2008). For example, animals breeding on the edge of a continent could use the coastline alongside a magnetic gradient to inform on position. However, such a mechanism is unlikely to work independent of topographic barriers to signal the end of migration. As an alternative, it has been suggested that single gradient cues could be used to inform on the position of the natal site by serving as a ‘stop sign’ on an otherwise pre-determined migratory bearing (Mouritsen 2003; Holland 2014). As with topography, using magnetic cues as a ‘stop sign’ on a migratory bearing would limit the impact of secular variation on the presumed position of the natal site and both could, therefore, be seen as a viable alternative to bi-coordinate information when considering the cues underlying philopatry.

Taken together, we believe our analyses not only outline the effects that secular variation could have on avian philopatry, but also make predictions as to where migratory birds should (or should not) return to if relying on magnetic information during natal/breeding site philopatry. We suggest, therefore, that the comparison of empirical data (e.g. ringing or tracking data) to predictions made using the IGRF may be of some considerable use when investigating philopatry. As with any simulation-led study, it is necessarily possible that our navigational models are too abstract to reflect the precise mechanisms by which birds navigate. Nonetheless, we believe our results may be informative when considering both the advantages and drawbacks of using different magnetic cues during avian philopatry.

## Supplementary Information

Below is the link to the electronic supplementary material.

Supplementary file1 (DOCX 8654 kb)
