# Supplementary material for: How might magnetic secular variation impact avian philopatry?
Source: J Comp Physiol A Neuroethol Sens Neural Behav Physiol. 2022 Feb 12;208(1):145–54. doi: 10.1007/s00359-021-01533-y (PMC8918480; doi:10.1007/s00359-021-01533-y)
Supplement: Supplementary file 1 — Supplementary file1 (DOCX 8654 kb) [file 359_2021_1533_MOESM1_ESM.docx]

**How might magnetic secular variation impact avian philopatry?**

*Variance in secular variation between regions*

Using the IGRF, we found that the sites represented by the intersect of specific magnetic isolines moved measurably between years. We found that the position denoted by the intersect of inclination/declination isolines moved a median average of 11.0km (± 0.204km [bootstrapped 95% CI]) between years, breaking down into a median distance of 9.17km (0.317km) for points in North America, 6.58km (0.0692km) in Asia and 24.2km (0.236km) in Europe (see figure 1). We found that the site represented by the intersect of specific declination/intensity isolines moved a median average of 21.4km (± 0.211km) between years, with points moving a median distance of 23.7km (0.221km) in North America, a median distance of 13.3km (0.375km) in Asia and a median distance of 25.0km (0.0692km) in Europe. Finally, we found that the site represented by the intersect of specific inclination/intensity isolines moved a median average of 28.4km (± 0.211km), breaking down into a median distance of 106.0km (1.67km) for points in North America, a median distance of 11.4km (0.323km) for points in Asia and a median distance of 30.9km (0.432km) for points in Europe (see figure 1).

*Supplementary figures*****

**Figure S1: Isoline positions for each of inclination, declination and intensity in the year 1900.** Isolines for focal magnetic parameters in (from left) Europe, Asia and North America. Points on the map represent randomly selected locations at which isolines were sampled for use in our analyis. Specific isoline values are included in figures S3-S5.

**Figure S2: Isoline positions for each of inclination, declination and intensity in the year 2000.** Isolines for focal magnetic parameters in (from left) Europe, Asia and North America. Points on the map represent randomly selected locations at which isolines were sampled for use in our analyis. Specific isoline values are included in figures S3-S5.

**Figure S3: Isoline positions for each of inclination, declination and intensity in the years 1900 and 2000 in Europe.** Isolines are shown for inclination (left), declination (centre) and intensity (right) , with the labels on each isoline denoting the value the isoline in question isoline represents**.**

**Figure S4: Isoline positions for each of inclination, declination and intensity in the years 1900 and 2000 in Asia.** Isolines are shown for inclination (left), declination (centre) and intensity (right), with the labels on each isoline denoting the value the isoline in question isoline represents**.**

**Figure S5: Isoline positions for each of inclination, declination and intensity in the years 1900 and 2000 in North America.** Isolines are shown for inclination (left), declination (centre) and intensity (right) , with the labels on each isoline denoting the value the isoline in question isoline represents**.**
